# Rapid mapping of the IAA in leaves of *Arabidopsis thaliana* using a simple paper-based electroanalytical device coupled with microsampling

**DOI:** 10.1039/d1ra03766h

**Published:** 2021-09-13

**Authors:** Ling Sun, Zhengfei Yang, Hao Li, Xiran Lan, Yishun Tang, Wu Liu, Xinyu Zhu, Ning Bao, Lijun Sun

**Affiliations:** School of Life Sciences, Nantong University Nantong Jiangsu 226019 China zhuxinyu@ntu.edu.cn slj.1226@163.com; National Key Laboratory of Plant Molecular Genetics, CAS Center for Excellence in Molecular Plant Sciences, Shanghai Institute of Plant Physiology and Ecology, Chinese Academy of Sciences 300 Fenglin Road Shanghai 200032 China; College of Life Sciences, Shanghai Normal University Shanghai 200234 China; School of Public Health, Nantong University Nantong Jiangsu 226019 China ningbao@ntu.edu.cn

## Abstract

To deeply investigate the pivotal roles of Auxin (mainly indole-3-acetic acid, IAA), it is essential to obtain the contents of IAA in different locations of plants. It is still a challenge to quantify the levels of IAA in different sites of *Arabidopsis thaliana* leaves because of the small sizes. In this study, a simple paper-based electroanalytical device coupled with microsampling was used to differentiate the IAA amounts in different locations of *Arabidopsis thalian*a leaves. For the micro real sampling, the different areas of the *thaliana* leaves were retrieved by the Harris Uni-Core TM Miltex® with diameters: 1.0, 1.5, 2.5, 3.5, and 4.0 mm. The results showed that the contents of IAA can be detected from circle samples with the diameter from 1.0 to 4.0 mm. With 1.5 mm diameter sampling, the levels of IAA could be obtained in different sites of cotyledon and the first true leaf of *Arabidopsis thaliana* at the seedling stage. Our results suggested that the highest IAA levels were in the near petiole and lowest IAA levels in the leaf tip, which roughly agreed with those in tobacco leaves based on HPLC-MS reported before. In addition, the microsampling has a minor impact on the growth of *Arabidopsis thaliana* in the following especially for circle samples with the diameter 1.5 mm. This study revealed the potential application of microsampling coupled with a simple paper-based electroanalytical device for the mapping study of IAA in small plants or small tissue samples.

## Abstract 2

To deeply investigate the pivotal roles of Auxin (mainly indole-3-acetic acid, IAA), it is essential to obtain the contents of IAA in different locations of plants.

## Graphical abstract

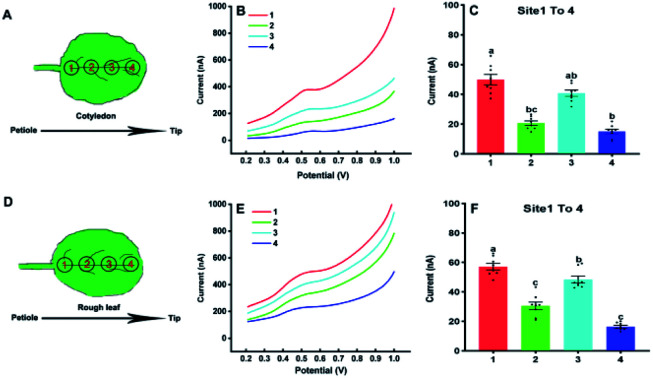

## Introduction

Auxin (mainly indole-3-acetic acid, or IAA) is probably one of the most important plant hormones, and interacts with other phytohormones to regulate growth and development of plants.^1–4^ The physiological functions of auxin strongly depend on its amounts in certain locations of plants, which are precisely and stably controlled. This process is called the homeostasis of auxin, which is accomplished based on biosynthesis, transportation and storage of auxin. Ljung *et al.*, showed that in the early stage of plant growth auxin is biosynthesized mainly in leaves and then transports to specific locations for its physiological functions.^5^

The key roles and properties of auxin in plants make it necessary to accurately quantify its real-time contents in different locations of plants, which is fundamental for investigation of the complex signal networks and the crosstalk between auxin and other plant hormones in plants. Most of plant hormones including auxin could be quantified using mass spectrometry after separation with gas chromatography or liquid chromatography.^6,7^ However, the time-consuming and complex steps for plant sample preparation make it difficult to determinate the auxin in real-time.^8^ More important, the instability of auxin also dues to its degrade during the complex sample preparation.^8^ Other techniques for analysis of auxin such as the enzyme-linked immunosorbent assay (ELISA) and the reporter genes^9–11^ also can be used to analyse the auxin. However, ELISA takes too much time to prepare antibodies with high costs while the expression effect is hard to be controlled for the reporter gene.

IAA is not stable because its indole ring could oxidized, which makes it possible to be electrochemically oxidized for its quantification. Carbon-based working electrodes (such as glassy carbon electrodes) were demonstrated to be effective for quantification of IAA.^12–14^ However, the settings of conventional electrochemical detection require the sample volume of around one milliliter, which limits their application for analysis of IAA in tiny plant samples.^12–14^ Recently, the paper-based analytical devices have been widely used to chemical and biochemical biological analysis.^15–24^ Compared with traditional approaches, the paper-based analytical device is superior because the sample volume for analysis could dramatically decrease to tens of microliters, which could release the pressure on the analytical sensitivity. We have demonstrated that electrochemical detection in paper-based analytical devices could be utilized for real time analysis of IAA and/or salicylic acid in pea seedlings and tomato fruits in our previous studies.^25–27^ By combining with the technique of micro-sampling, such decrease not only facilitated the analysis of IAA in real plant samples but also enhanced the analytical performance.^28^

*Arabidopsis thaliana* has become a popular model plant in the study of plant science for decades.^29^ In *Arabidopsis thaliana*, there are about 125-megabase genome that could be classified into five chromosomes. In a common lab, *Arabidopsis thaliana* could experience the growth stages of sprout, seeding, vegetative, budding, flowering and ripening within just 3 months. With such a short period, one individual *Arabidopsis thaliana* could generate about one thousand seeds. More importantly, the technique of floral tip offers a simple and effective approach for gene modification of *Arabidopsis thaliana*. For those reasons, a lot of information obtained from *Arabidopsis thaliana* has been accumulated, which significantly facilitate the study of plant science. However, there is still no satisfactory way to obtain the distribution of IAA in individual *Arabidopsis thaliana* leaves although this is critical for the study of the functions of IAA. This is because *Arabidopsis thaliana* is much smaller. For example, the area of single leaves is around 20 square millimeters at the seedling stage. Currently the technique of VENUS could be used to address this problem, but its application was limited by its complicated steps and expensive instruments.

In this paper, the contents of IAA in *Arabidopsis thaliana* leaves were screened using paper-based electroanalytical devices and the technique of microsampling. Compared with our previous studies,^25–27^ the sampling area of *Arabidopsis thaliana* leaves could reach 1 square mms for detection of IAA based on electrochemical detection. Then the levels of IAA could be obtained in different sites of *Arabidopsis* leaves. The influences of the damage brought by the microsampling on following growth were also investigated. This study provided a novel method to study the mapping of IAA in small plants or tiny tissue.

## Materials and methods

### Chemicals and materials

The chemicals of indole-3-acetic acid (IAA), sucrose and MES at the analytical grade were purchased from Sigma-Aldrich (St. Louis, MO, USA). Agar was from Biosharp Co. Ltd. (Hefei, Anhui, China). The samples (diameters: 1.0, 1.5, 2.5, 3.5, and 4.0 mm) of Harris Uni-Core TM Miltex® were obtained from Ted Pella, Inc (Redding, California, USA). The Indium tin oxide (ITO) conductive glass (355.6 mm wide, 406.4 mm long, 1.1 mm thick, STN, 10 Ω) was purchased from Nanbo Display Technology Co. LTD. (Shenzhen, China). The conductive double-sided carbon adhesive tape (8 mm wide, 0.16 mm thick and 20 m long) was from SPI Supplies (West Chester, PA, USA). The qualitative filter paper (Whatman No. 1) was purchased from Whatman International Ltd. (Maidstone, UK). The Phosphate buffer (PB) solution (0.2 M) was prepared by mixing Na_2_HPO_4_ and NaH_2_PO_4_ with the pH modified to be 7.4. In all experiments the double distilled water was used.

### Culture of *Arabidopsis thaliana*

The seeds of wild type (Col-0) *Arabidopsis thaliana* were treated with 75% alcohol for 20 min and then repeatedly washed with sterilized water for four times. The washed seeds were kept at 4 °C for 2 days and then placed in 1/2 MS medium (half-strength of MS basal medium with 1% sucrose, 1% agar, and 0.5 g L^−1^ MES, pH 5.8) for germination. After that, *Arabidopsis thaliana* seedlings were transplanted in 4 : 1 vermiculite and nutritious soil, cultured at 22 °C, illuminated for 16 h (∼5000 Luxu, cold white fluorescent lamp), dark light cycle for 8 h, and cultured in plant tissue incubator for around 21 days until the first true leaf appears. *Arabidopsis* cotyledons growing for 21 days were chosen as the sampling leaves for analysis of IAA.

### Construction of paper-based electroanalytical devices

The fabrication of the modified carbon tape electrodes has been reported previously.^25^ Briefly, a piece of carbon tape (8 mm long and 7 mm wide) was attached on a piece of the conductive ITO glass (20.0 mm long and 7 mm wide) and then modified with 15 μL 0.025 mg mL^−1^ multi-wall carbon nanotubes (150 μL, 2 mg mL^−1^ multi-wall carbon nanotubes and 850 μL water). A piece of transparent tape with a hole was applied on the dried carbon tape to provide effective area for electrochemical detection.^25^ The modified electrodes were treated under oxygen plasma for 1 min in a PDC-32 G plasma cleaner (Harrick Plasma, Ithaca, NY). Then a piece of circular filter paper was applied on the modified electrodes for detection.^25^ It needs to emphasize the filter paper could not only store the buffer solution but also provide conductive connection among the electrodes.

### Microsampling and multichannel electrochemical detection

**Scheme 1:**
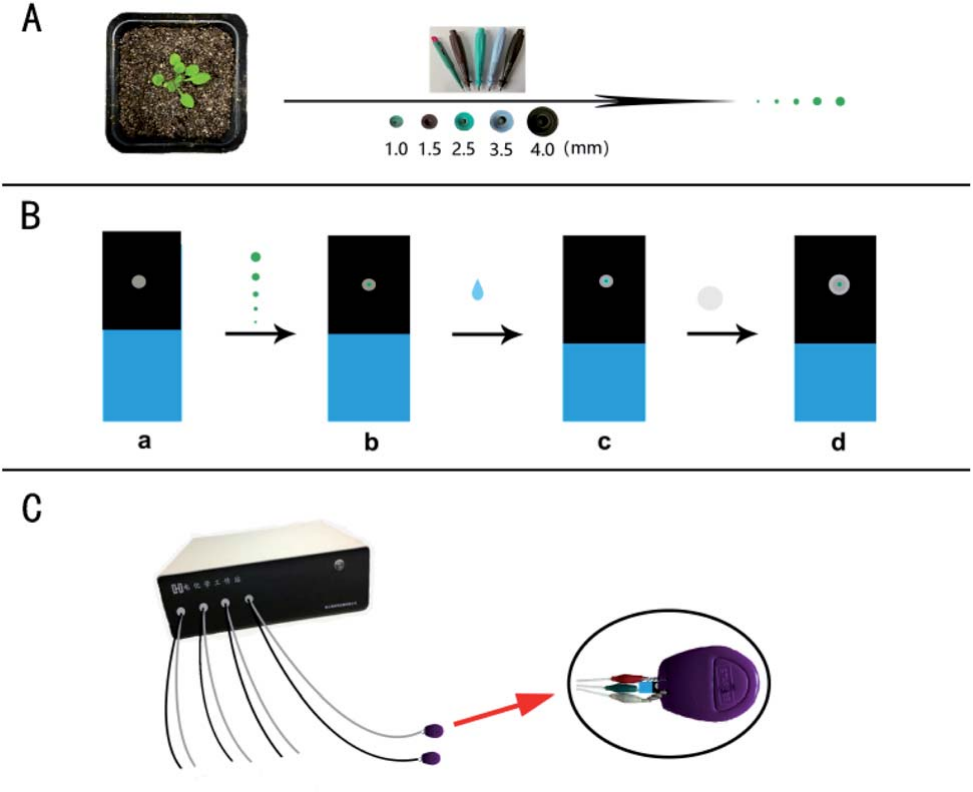
(A) For the micro real sampling, the different area of the plant sample was retrieved by the Harris Uni-Core TM Miltex® with the diameters: 1.0, 1.5, 2.5, 3.5, and 4.0 mm. (B) The working electrode, ITO conductive glass was presented by blue, and carbon tape affixed on the ITO conductive glass presented by black (a). The obtained plant samples with the different areas were put on the surface of the working electrode (b). The PB solution with the volume of 10 μL was dropped on the surface of the working electrode (c), and finally a piece of filter paper was used to cover the electrode surface (d). (C) A clasp with a platinum wire and an Ag/AgCl wire for electrochemical detection based on a multi-channel (8-channel) electrochemical station.

For the micro real sampling, the different area of the samples was retrieved by the Harris Uni-Core TM Miltex® with the diameters: 1.0, 1.5, 2.5, 3.5, and 4.0 mm (Scheme 1A). The retrieved samples with the different diameters were placed on the surface of a working electrode for detection (Scheme 1B). Differential pulse voltammetry (DPV) was performed on a multi-channel CHI1040C electrochemical workstation (Scheme 1C). The carbon tape modified electrode was used as the disposable working electrode, which means a new electrode was used for every analysis. The Ag/AgCl wire was used as the reference electrode and platinum wire as the counter electrode. The potential is from 0.2 to 1.4 V. Other parameters are: 0.1 V increase potential, 0.05 V amplitude, 0.2 s pulse width, 0.0667 s sampling width, 0.1 s pulse period, 20 s equivalent time. The sample of the standard solution or the plant sample (with 10 μL PB) was put on the working electrode for analysis of IAA by covering with a piece of the filter paper. Before each test, the counter electrode and the reference electrode were cleaned thoroughly with double distilled water. If necessary, the DPV curve is processed by baseline subtraction and peak splitting in the origin software (Northampton, MA, USA). By fitting the Gaussian peak, the IAA peak is obtained. Typical DPV curves of IAA in plant sample with the different area were obtained based on a multichannel electrochemical station (Scheme 1C).

## Results and discussion

**Fig. 1:**
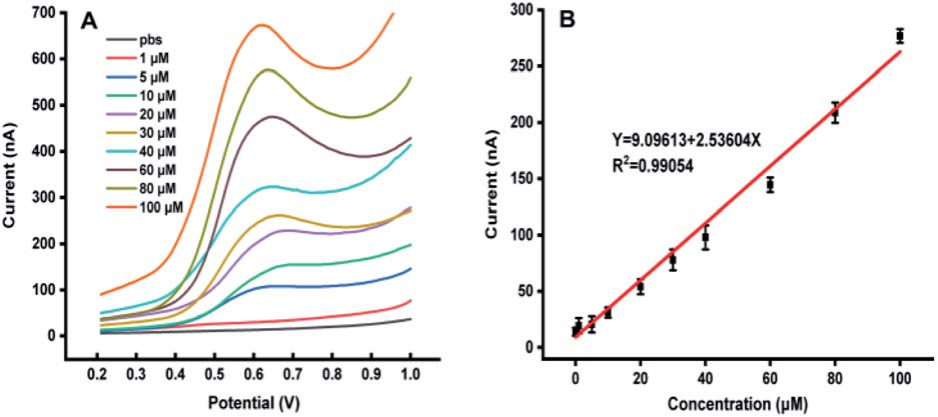
(A) The DPV curves with the different concentration of IAA. (B) The relationship between the peak heights with the concentration of IAA, all experiments were repeated eight times. Data are shown as means ± SEM.

Fig. 1 shows the dependence of electrochemical responses of IAA on the concentration at the carbon tape modified electrodes integrated in paper-based analytical devices. It could be found that with the increase of the IAA concentration the peak heights increased accordingly. It should be emphasized that the high concentration of IAA made the pH decreasing, which leaded to deviation in the position of the oxidation peak. The linear relationship between the electrochemical responses and the concentration of IAA indicated that our approach could be used for quantification of IAA in plants.

**Fig. 2:**
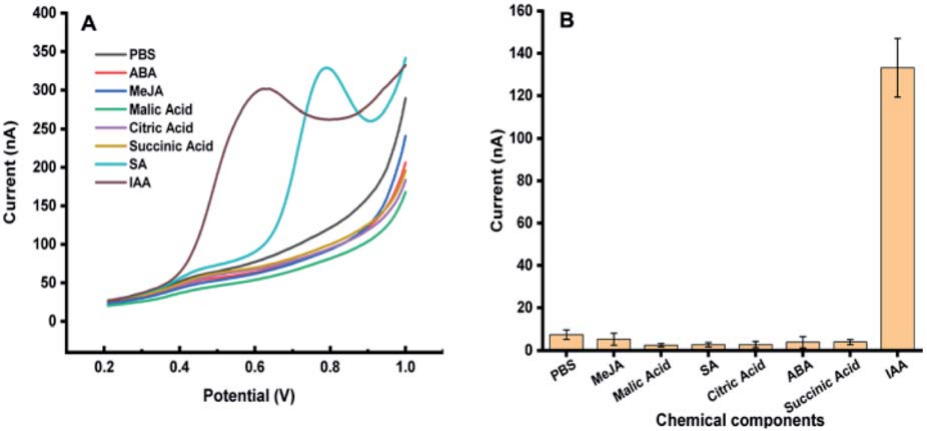
Electrochemical response of 50 μM methyl jasmonate (MeJA), malic acid, salicylic acid (SA), citric acid, succinic acid, abscisic acid (ABA), and indole-3-acetic acid (IAA) on carbon band modified electrode. (A) DPV curves of different components in the plant. (B) Eight repeated measurements of different plant signal molecules, and the average value and standard deviation were obtained. Experimental parameters: the same as those in Fig. 1.

The possible interferences from the components in plant samples such as citric acid, malic acid, succinic acid, abscisic acid, methyl jasmonate and salicylic acid were also studied. It was observed that all the above components had no electrochemical response for the detection of IAA except salicylic acid (Fig. 2). Although salicylic acid has a peak at the potential of about 0.78 V, it does not overlap with the potential of IAA. These results showed that the existence of these components will not affect the detection of IAA in our system. In addition, the repeatability of the MWCNTs/Nafion modified carbon tape electrodes was tested to be 7%.

**Fig. 3:**
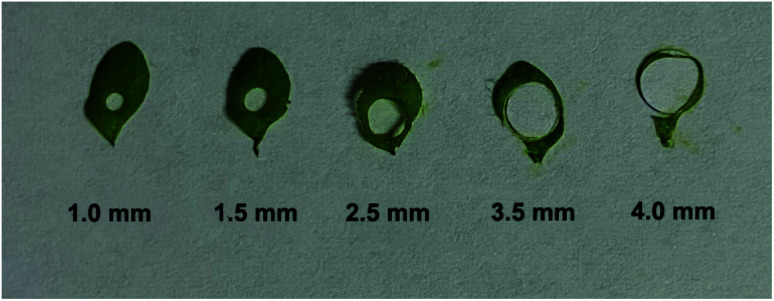
The sampling area on individual *Arabidopsis thaliana* leaves.

**Fig. 4:**
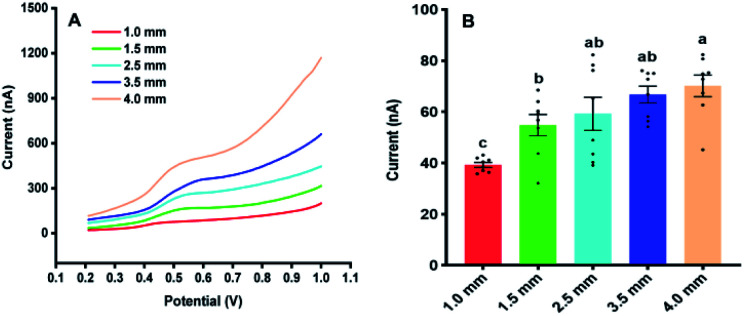
The Influences of the sampling area on the electrochemical responses of IAA in *Arabidopsis thaliana* leaves. (A) The DPV curves of IAA in plant samples with different areas. (B) The contents of IAA in the plant samples with the different areas. All experiments were repeated eight times. Data are shown as means ± SEM. Bars with different letters indicate significant differences at *p* <0.05 by two-way ANOVA with Tukey's multiple comparison test.

Fig. 3 illustrates the sampling of *Arabidopsis* leaves at the seedling stage with different areas. It was found that with the sample area of 4 mm diameter, almost all the leaf was used for the analysis. With the diameter of the sample area decreased from 4 to 1 mm, more and more areas of the *Arabidopsis* leaves were maintained. Fig. 4 shows the electrochemical responses of IAA in the samples with different area. The typical DPV curves of IAA in plant samples with different areas were showed in Fig. 4A. Fig. 4B showed the peak height of the IAA and all the data points. It was found that with the increase of the diameter of the samples, the peak heights of IAA significantly increased. It is worth noting that even with the diameter of 1 mm, IAA could still be differentiated. These results showed that our approach could be used to detect the IAA in the small plants or tiny tissue.

**Fig. 5:**
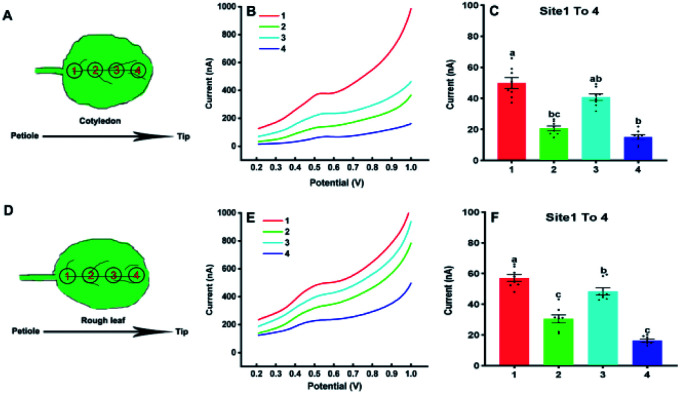
Analysis of IAA in four sites of cotyledons (A) and the first true leaves (D) using the paper-based electroanalytical devices; DPV curves (B) and the IAA contents (C) in four sites of cotyledons; DPV curves (E) and the IAA contents (F) in four sites of the first true leaves of *Arabidopsis thalian*a in 21 days. All experiments were repeated eight times. Data are shown as means ± SEM. Bars with different letters indicate significant differences at *p* <0.05 by two-way ANOVA with Tukey's multiple comparison test.

In the following, our approach was utilized for differentiation of IAA in four sites of the cotyledon and the first true leaf (rough leaf) of *Arabidopsis* (Fig. 5). The *thaliana* leaves was retrieved by the Harris Uni-Core TM Miltex® with diameters 1.5 mm. The results showed fluctuated electrochemical responses of IAA in different sites. It could be seen that there was significant difference between site 1 and 2, site 1 and 4 in the cotyledon (Fig. 5C). For the first true leaf, there were significant differences among the different sits (Fig. 5F). In both cotyledon and the rough leaf, the levels of IAA are the highest in site of 1 and lowest in site of 4, which suggested that the highest IAA levels in the near petiole and lowest IAA levels in the leaf tip. It could also be found that the total IAA amounts in four locations in the rough leaf were higher than those in cotyledon. The varied IAA amounts in *Arabidopsis* leaves roughly agreed with those in the tobacco leaf using gas chromatography and mass spectrometry.^30^ It needs to be emphasized that the length of the *Arabidopsis* leaves is about 6 mm, which is much smaller than that of tobacco leaves.

**Fig. 6:**
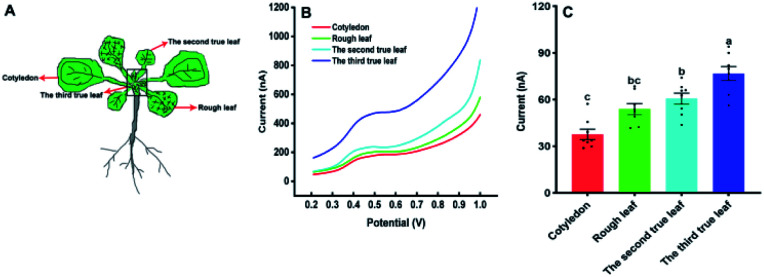
The amount of IAA in the different kind of *Arabidopsis thaliana* leaves. (A) The leaf sequences of *Arabidopsis thaliana*; (B) the DPV curves of IAA content in different leaf sequences of *Arabidopsis thaliana*. (C) IAA content in different leaf sequences of *Arabidopsis thaliana*. All experiments were repeated eight times. Data are shown as means ± SEM. Bars with different letters indicate significant differences at *p* <0.05 by two-way ANOVA with Tukey's multiple comparison test.

Our approach was also used to evaluate the amounts of IAA in different types of *Arabidopsis* leaves with the diameter 4 mm. Fig. 6 shows the IAA content of cotyledons, rough leaves, the second true leaf and the third true leaf. From Fig. 6C, it can be found that the level of IAA in cotyledons is the lowest, while the level of IAA in the third true leaf is the highest. The results suggested that the highest relative synthesis capacity of IAA in the youngest developing leaves. Our results are also in good agreement with the previously reported results based on gas chromatography and mass spectrometry.^30^

**Fig. 7:**
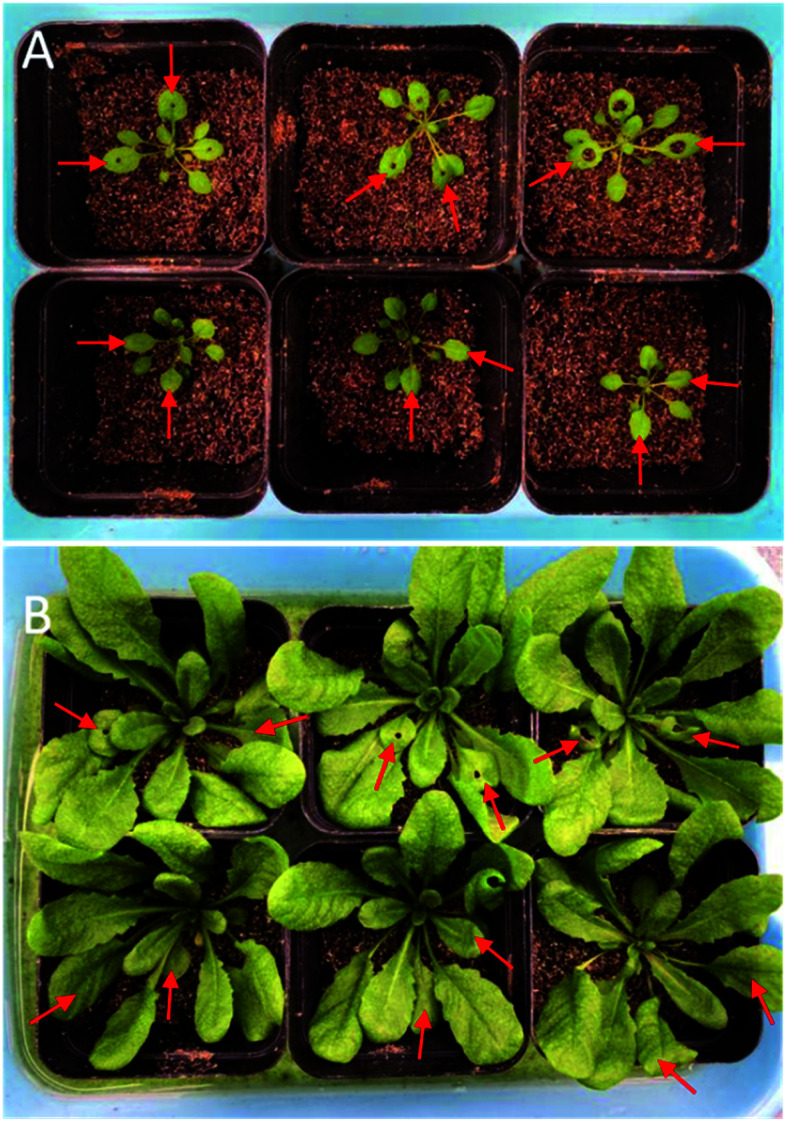
The influence of microsampling of *Arabidopsis* leaves on their following growth. (A) The leaves of *Arabidopsis thaliana* were perforated with samplers (1.5, 2.5 and 4.0 mm) at the age of 2 weeks. (B) The growth status of *Arabidopsis thaliana* at the age of 4 weeks after microsampling. The red arrows showed the age of leaves were similar between the wounding and control.

It is worthy to note that our approach is still invasive although the sampling area could decrease to a circle with the diameter of 1 mm. Therefore, the influences of the damage brought by the microsampling on following growth were also investigated. As shown in Fig. 7A, the leaves of *Arabidopsis thaliana* were perforated with the samplers (1.5, 2.5 and 4.0 mm) at the age of 2 weeks. Within 2 weeks after micro-sampling, the size and the growth status of the *Arabidopsis* leaves with the sample area of 1.5 and 2.5 mm diameter was not significantly difference with the same age leaves of the control (Fig. 7B). In addition, the *Arabidopsis* grew well after fifteen days even with the sampling area of 4 mm diameter (Fig. 7B). Such results implied that the technique of micro-sampling would have no insignificant impact on the growth of *Arabidopsis*, especially for the microsampler with the 1.5 mm diameter.

## Conclusions

IAA is one of the most important plant hormones, playing an important role in the whole cycle of plants. Leaves are the main organs of plants for photosynthesis and respiration, significantly affecting the growth state of plants. Herein a simple paper-based electroanalytical device coupled with microsampling technique was used to study the IAA amounts in *Arabidopsis thaliana* leaves. Our results showed that the contents of IAA can be detected from circle samples with the diameter from 1.0 to 4.0 mm, which minor impact on the growth of *Arabidopsis thaliana* in the following, especially for the diameter 1.5 mm. The levels of IAA could be obtained in different sites of leaves of *Arabidopsis thaliana* at the seedling stage, which suggested that the highest IAA levels in the near petiole and lowest IAA levels in the leaf tip. In addition, our results also suggested that the highest relative synthesis capacity of IAA in the youngest developing leaves. Our study implied that the paper-based electroanalytical devices coupling the microsampling technique might be widely applied for the study of IAA amounts in different locations of living plants, epically for small plants or tiny tissue.

## Author contributions

L. S., Z. Y. and H. L. for the data collection, data analysis, and figures. X. L., Y. T. and W. L. for data interpretation and figures. X. Z. and N. B. for writing. L. S. for the study design, literature search, data analysis, data interpretation, and writing. All authors have read and agreed to the published version of the manuscript.

## Conflicts of interest

There are no conflicts to declare.

## Supplementary Material
